# Low-intensity low-frequency pulsed ultrasound ameliorates sciatic nerve dysfunction in a rat model of cisplatin-induced peripheral neuropathy

**DOI:** 10.1038/s41598-022-11978-z

**Published:** 2022-05-17

**Authors:** Busra Bilir-Yildiz, Fatma Bahar Sunay, Hatice Fulya Yilmaz, Ozlem Bozkurt-Girit

**Affiliations:** 1 Department of Biophysics, School of Medicine, Aydın Adnan Menderes University, Aydın, 09010 Turkey; 2 Department of Histology and Embryology, School of Medicine, Balıkesir University, Balıkesir, Turkey

**Keywords:** Biophysics, Neurology, Neurological disorders

## Abstract

Chemotherapy-induced peripheral neuropathy is a neurological complication that frequently occurs during chemotherapeutic intervention, resulting in damaged myelin sheath, motor weakness and/or sensory impairment. This study aims to investigate the therapeutic efficiency of low-intensity pulsed low-frequency ultrasound on cisplatin-induced peripheral neuropathy. Rats were randomly divided into five experimental groups as control, cisplatin administration, 10 mg/kg melatonin treatment after cisplatin administration, 1 MHz frequency 0.5 W/cm^2^ pulsed ultrasound treatment after cisplatin administration and 1 MHz frequency 1.5 W/cm^2^ pulsed ultrasound treatment after cisplatin administration. Chemical neuropathy was induced by the injection of 3 mg/kg/week of cisplatin (i.p.) for 5 weeks. Afterwards, melatonin and pulsed ultrasound treatments were applied for 15 consecutive days. Cisplatin administration resulted in a decrease in nociceptive pain perception and nerve conduction velocities together with a decrease in myelin thickness and diameters of axons and myelinated fibers, indicating a dysfunction and degeneration in sciatic nerves. In addition, cisplatin administration led to a decrease, in superoxide dismutase activity, and an increase in malondialdehyde and IL-1β levels together with an increase in caspase-3 protein expression levels and a decrease in Bcl-2 and Parkin levels. The ultrasound treatments resulted in an increase in nociceptive pain perception and sciatic nerve conduction; led to a decrease in oxidative stress and inflammation, restored nerve degeneration and regulated apoptosis and mitophagy. Taken together, low-intensity pulsed low-frequency ultrasound was efficient in restoring the alterations attributable to cisplatin-induced peripheral neuropathy, and warrants further investigations.

## Introduction

Peripheral neuropathy can be defined as the deterioration of the structure of peripheral nerves that results in the loss of deep tendon reflex, sensory nerve dysfunction, motor and muscle weakness^1^*.* Damage to the involved nerves may manifest mainly in the form of axonal degeneration, segmental demyelination, or a combination of both. In addition, peripheral neuropathy may result in excessive myelin loss and eventually loss of axons^2^. There are a number of factors that can lead to peripheral neuropathy including diabetes, inherited diseases, poor nutrition, certain medications and chemotherapeutic agents^3^. The type of peripheral neuropathy is named as chemotherapy-induced neuropathy when the damage is caused by the exposure of toxic chemotherapeutic agents.

Cisplatin is the first synthesized molecule among platinum-based anti-cancer drugs and is one of the most important antineoplastic drugs used in cancer treatment. It is used in the treatment of solid tumors, such as lung, testis, head, neck, bladder and kidney cancers and lymphoma with hematological cancer^4,5^. Cisplatin interacts with DNA, causing cross-linking in the chain and creating openings in the DNA helix, thus preventing cell proliferation and demonstrating cytotoxic effect on tumors and rapidly dividing cancerous cells^6–8^.

Cisplatin-induced peripheral neuropathy usually appears with axonal damage, which manifests itself after a period following cisplatin administration^9^. It has been reported in many studies that neuropathy induced either by chemotherapy or any other factors causes a decrease in mitochondrial function due to increased reactive oxygen species and oxidative stress in nerve cells^10,11^. Moreover, recent scientific advances have implicated that the molecular mechanism of neurodegeneration and neuropathy may be related to the inhibition of autophagy and mitophagy leading to the accumulation of oxidant damage in proteins and organelles. Therefore, methods to regulate autophagy or mitophagy were suggested to play an important role in the treatment of peripheral neuropathy by the reduction of nerve damage^11–13^.

It has been suggested that ultrasound (US) has a therapeutic effect in tissue repair processes by stimulating cell proliferation and differentiation^14^. Indeed, low-intensity pulsed US has become a routine practice in the treatment of pseudoarthrosis, bone fractures and soft tissue healing. Recently, Wang et al*.*^15^ showed that low-intensity pulsed US promotes chondrogenesis by regulating autophagy in mesenchymal stem cells. Low-intensity pulsed US application was reported to accelerate nerve regeneration in animal models of peripheral nerve injury, increasing the proliferation of human and animal Schwann cells, and enhancing various gene and protein expressions of neural race^9,16^. However, the responses obtained with the US treatment vary depending on the power density, frequency, and duration of the US ^15^. Yet, there is no consensus on the values of these parameters in the reported studies in the literature. In addition, the efficacy of US treatment in chemotherapy-induced peripheral neuropathy has not yet been investigated. Accordingly, this study was conducted to assess the efficacy of low-intensity pulsed low-frequency US in the treatment of cisplatin-induced peripheral neuropathy in rats.

## Results

### Nociceptive pain perception

**Figure 1:**
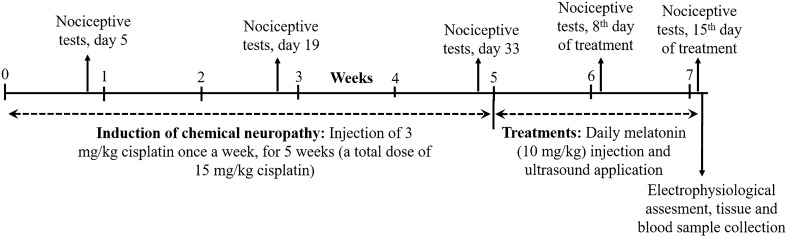
Schematic diagram demonstrating the timing of experimental procedures applied on rats.

Figure 1 demonstrates the timing of the applied experimental procedures. According to the nociceptive test results, during the 5-week period of cisplatin injection, there was a considerable increase in the latencies of the reactions given in response to thermal painful stimuli in comparison to the control group (Table 1), which revealed the decreased pain perception upon cisplatin administration. All treatments resulted in a significant decrease in nociceptive hot plate and tail flick latencies (*p* < 0.05) in comparison to the cisplatin-induced neuropathy group by the end of the treatment period (Table 2).

**Table 1:** Results of the nociceptive tests recorded as latency (s) of a reaction given in response to thermal painful stimuli during the 5-week period of chemical neuropathy induction.

Nociceptive test results	Latency (s)			
	Groups	1^st^ week	3^rd^ week	5^th^ week
Hot Plate	C	4.33 ± 0.18	3.57 ± 0.16	4.19 ± 0.11
	CP	5.26 ± 0.09**	5.68 ± 0.10**	5.62 ± 0.08***
Tail Flick	C	4.99 ± 0.11	4.57 ± 0.17	4.75 ± 0.13
	CP	6.06 ± 0.09**	6.19 ± 0.08***	6.26 ± 0.07***

The data were represented as mean ± standard error of mean. Differences in between variances were compared by One-way ANOVA test with Tukey’s test applied as a post-hoc test. *P*-values equal or less than 0.05 were considered as statistically significant (*p < 0.05, **p < 0.01, ***p < 0.001). The degree of significance was symbolized with asterisks (*) for the comparisons to that of control group (C).

**Table 2:** Results of the nociceptive tests recorded as latency (s) of a reaction given in response to thermal painful stimuli during the 15-day treatment period.

Nociceptive Tests	Groups	Latency (s)		
		Before treatment	Mid-treatment	End of treatment
Hot-plate	C	4.60 ± 0.18	4.83 ± 0.17	4.86 ± 0.15
	CP	5.97 ± 0.18	5.86 ± 0.21*	5.81 ± 0.11*
	CP + MEL	5.10 ± 0.14	4.21 ± 0.11*	4.01 ± 0.09*^, ‡^
	CP + US (0.5 W/cm^2^)	5.33 ± 0.21	3.97 ± 0.16*	3.64 ± 0.10*^, ‡^
	CP + US (1.5 W/cm^2^)	5.20 ± 0.16	3.96 ± 0.16*	3.21 ± 0.17*^, ‡^
Tail-flick	C	4.87 ± 0.12	4.45 ± 0.25	4.90 ± 0.12
	CP	6.57 ± 0.15	6.72 ± 0.29*	7.25 ± 0.48*
	CP + MEL	6.23 ± 0.15	4.18 ± 0.21*	3.88 ± 0.10**^, ‡^
	CP + US (0.5 W/cm^2^)	6.24 ± 0.22	3.81 ± 0.24*	3.72 ± 0.19**^, ‡^
	CP + US (1.5 W/cm^2^)	5.88 ± 0.21	3.69 ± 0.17*	3.42 ± 0.16**^, ‡^

The data were represented as mean ± standard error of mean. Differences in between variances were compared by One-way ANOVA test with Tukey’s test applied as a post-hoc test. *P*-values equal or less than 0.05 were considered as statistically significant (*p < 0.05, **p < 0.01, ***p < 0.001). The degree of significance was symbolized by an asterisks (*) for the comparison of all groups with respect to the control group; by a double daggers (‡) for the comparison of the treatment groups with respect to the cisplatin administered group; by a daggers (†) for the comparison of ultrasound treated groups with respect to the melatonin administered group and by a section (§) for the comparison of 1 MHz 1.5 W/cm^2^ US treated group with respect to the 1 MHz 0.5 W/cm^2^ US treated group.

### Electrophysiological recordings

Table 3 demonstrates the alterations in the motor nerve conduction velocities, distal latencies, peak-to-peak amplitude, area and duration of the compound muscle action potential (CMAP) recordings of all experimental groups. As can be seen from Table 3, cisplatin administration led to a significant decrease in motor nerve conduction velocities (MNCV) in comparison to that of the control group (*p* < 0.001). The treatments resulted in an increase in sciatic MNCV in comparison to that of cisplatin-induced neuropathy group (*p* < 0.05). In addition, there was a significant decrease in the amplitudes of the CMAP recordings (*p* < 0.01) of cisplatin administered group compared to the control group (Table 3). This finding demonstrated the presence of axonal damage in cisplatin-induced neuropathy group, as axonal damage and demyelination results in the decrease in amplitude values in CMAP recordings^17,18^. The duration of the recorded CMAP was observed to slightly increase in the cisplatin-induced neuropathy group, whereas it was significantly decreased in the US treatment groups (*p* < 0.05).

**Table 3:** The results of the alterations in distal latency, peak-to-peak amplitude, area and duration of the compound muscle action potential recordings, and the sciatic motor nerve conduction velocities in the experimental study groups.

Electophysiological measurements	Groups				
	C	CP	CP + MEL	CP + US (0.5 W/cm^2^)	CP + US (1.5 W/cm^2^)
Motor nerve conduction velocity (m/s)	76.65 ± 0.43	38.07 ± 0.32***	54.92 ± 0.24**^,‡^	51.98 ± 0.12**^,‡^	50.25 ± 0.14**^,‡^
Distal latency (s)	0.0025 ± 0.0010	0.0030 ± 0.0011	0.0023 ± 0.0020	0.0022 ± 0.0013	0.0022 ± 0.0010
Amplitude (peak-to-peak) (mV)	10.18 ± 0.23	3.91 ± 0.39**	5.46 ± 0.21*	6.57 ± 0.10^‡^	7.19 ± 0.33^‡^
Area (mV.ms)	0.0027 ± 0.0004	0.0020 ± 0.0001	0.0017 ± 0.0001	0.00016 ± 0.0002	0.00017 ± 0.0001
Duration (ms)	1.02 ± 0.22	1.86 ± 0.24	0.87 ± 0.05	0.70 ± 0.06*^,‡^	0.66 ± 0.09*^,‡^

The data were represented as mean ± standard error of mean. Differences in between variances were compared by One-way ANOVA test with Tukey’s test applied as a post-hoc test. *P*-values equal or less than 0.05 were considered as statistically significant (*p < 0.05, **p < 0.01, ***p < 0.001). The degree of significance was symbolized by an asterisks (*) for the comparison of all groups with respect to the control group; by a double daggers (‡) for the comparison of the treatment groups with respect to the cisplatin administered group; by a daggers (†) for the comparison of ultrasound treated groups with respect to the melatonin administered group and by a section (§) for the comparison of 1 MHz 1.5 W/cm^2^ US treated group with respect to the 1 MHz 0.5 W/cm^2^ US treated group.

### Western blot analysis

**Figure 2:**
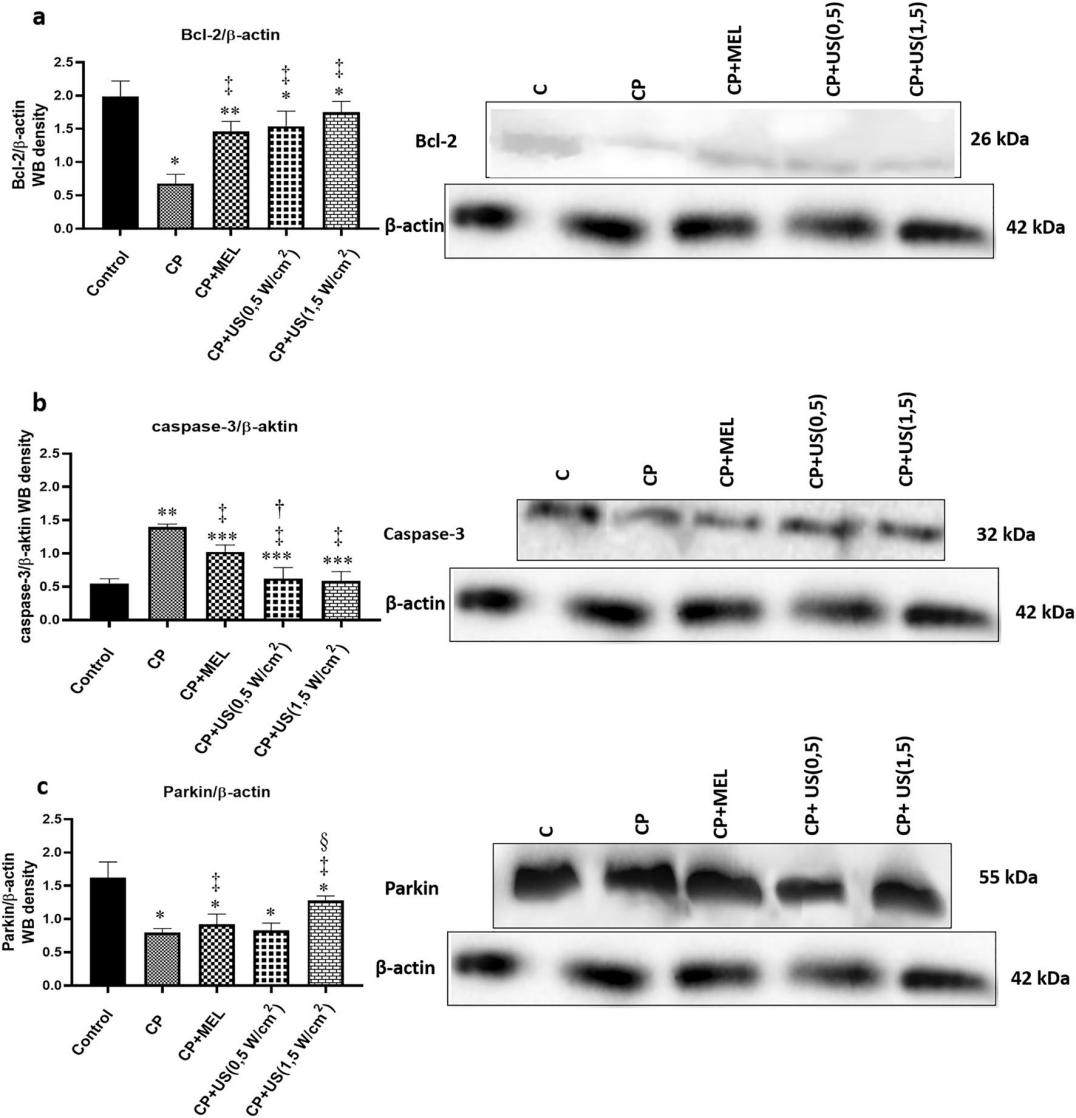
Results representing the alterations in protein expression levels. Representative Western blot images and the calculated protein band intensity results of the expression levels of (**a**) anti-apoptotic marker Bcl-2, (**b**) pro-apoptotic marker caspase-3 and (**c**) marker of mitophagy Parkin proteins, analyzed by ImageJ compared to that of β-actin. The represented Western blot images for each protein investigated were cropped from different blots, where same exposure was used for visualization. The full-length blots can be seen in Supplementary Fig. S1. The samples were derived from the same experiment and the blots were processed in parallel. C, control group; CP, cisplatin administered group; CP + US 0.5 W/cm^2^, cisplatin administered and 0.5 W/cm^2^ US treated group; CP + US 1.5 W/cm^2^, cisplatin administered and 1.5 W/cm^2^ US treated group; CP + MEL, cisplatin administered and melatonin treated group. Data were represented as mean ± SEM. The degree of significance was symbolized by an asterisks (*) for the comparison of all groups with respect to the control group; by a double daggers (‡) for the comparison of the treatment groups with respect to the cisplatin administered group; by a daggers (†) for the comparison of ultrasound treated groups with respect to the melatonin administered group and by a section (§) for the comparison of 1 MHz 1.5 W/cm^2^ US treated group with respect to the 1 MHz 0.5 W/cm^2^ US treated group (*p < 0.05, **p < 0.01, ***p < 0.001).

The expression levels of Bcl-2 and caspase-3 proteins in the sciatic nerve tissue homogenates were determined to provide information about the activation of apoptotic pathway, and the expression level of Parkin protein was examined to provide information about the induction of mitophagy. The obtained results were analyzed in comparison to that of β-actin. As seen in Fig. 2a, the expression level of Bcl-2/β-actin was significantly decreased in the cisplatin administered group compared to the control group (*p* < 0.05), whereas a significant increase in Bcl-2 expression level was observed in melatonin, 1 MHz 0.5 W/cm^2^ US and 1 MHz 1.5 W/cm^2^ US treatment groups compared to the control group and cisplatin group (*p* < 0.05). The increase was more pronounced in US-treated groups, especially in the 1 MHz 1.5 W/cm^2^ US treatment group.

Figure 2b shows the alterations in the caspase-3/β-actin protein expression levels in sciatic nerves. As can be seen from the figure, the level of caspase-3 protein was significantly higher in cisplatin administered group in comparison to that of control (*p* < 0.01). Upon treatments, the level of caspase-3 was significantly decreased (*p* < 0.05) in comparison to cisplatin administered group, where the decrease in the caspase-3 expression level was significantly lower in 1 MHz 0.5 W/cm^2^ US treatment group (*p* < 0.05) in comparison to that of melatonin administration.

Figure 2c shows the protein expression levels of Parkin/β-actin in the experimental groups. Parkin is a protein that takes role in the ubiquitination of mitochondrial outer membrane proteins and is known to play an important role in the destruction of the damaged mitochondria^19^; therefore, gives information about the induction of mitophagy. As can be seen in Fig. 2c, the expression level of Parkin protein was significantly decreased in cisplatin administered group (*p* < 0.05) in comparison to that of control. The levels of Parkin protein were observed to be similar to that of cisplatin administered group in 1 MHz 0.5 W/ cm^2^ US treated group; however, there was a significant increase in Parkin protein expression level in melatonin administered and 1 MHz 1.5 W/cm^2^ US treatment group (*p* < 0.05) in comparison to that of cisplatin administered group. This finding implies that 1 MHz 1.5 W/cm^2^ US treatment led to an improvement in the induction of mitophagy.

### Biochemical results

**Figure 3:**
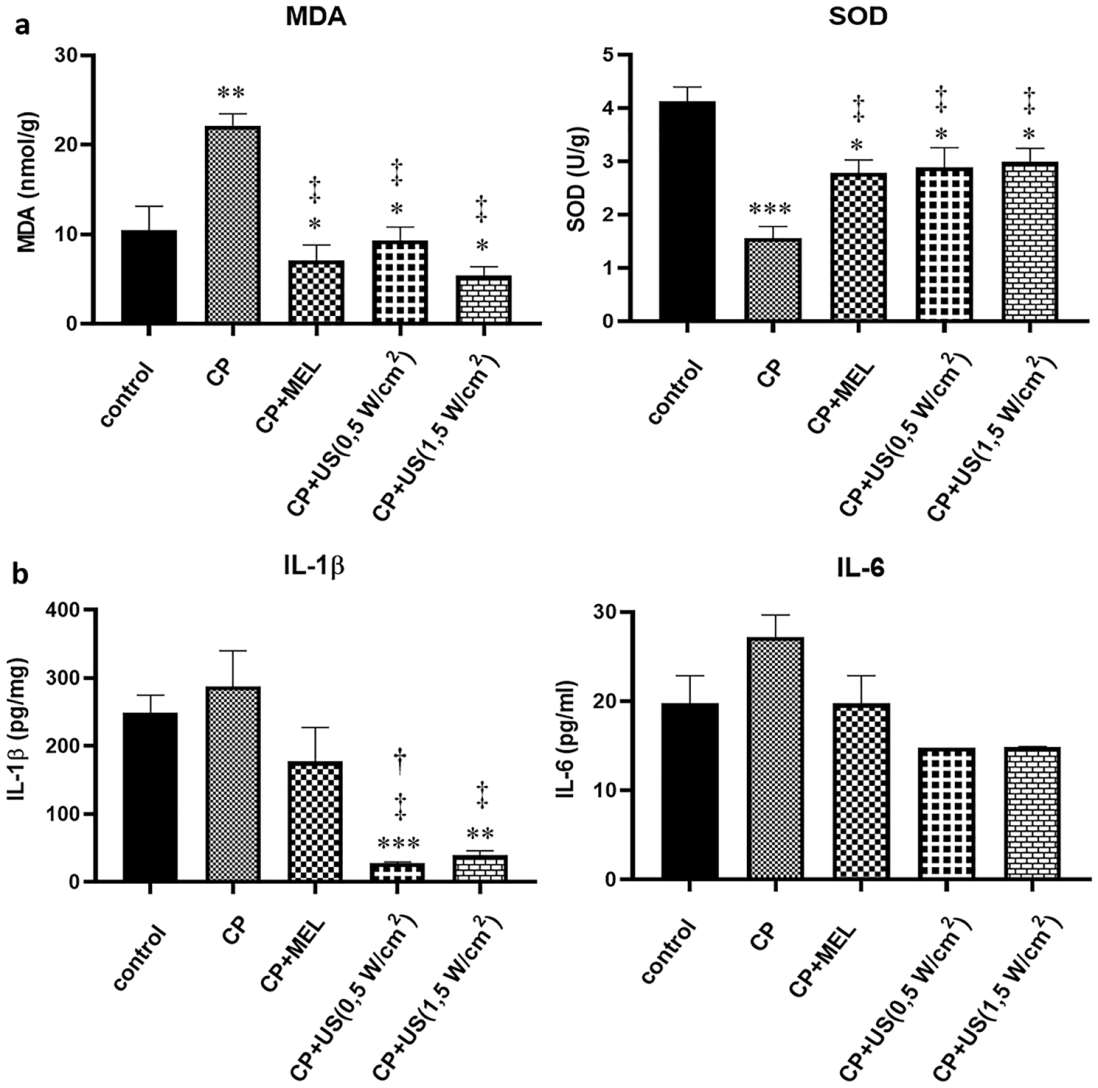
Results representing alterations in oxidative stress and inflammation. Bar diagrams representing MDA levels and SOD activity in sciatic nerve tissue samples (**a**), and serum IL-1β and IL-6 levels (**b**). C, control group; CP, cisplatin administered group; CP + US 0.5 W/cm^2^, cisplatin administered and 0.5 W/cm^2^ US treated group; CP + US 1.5 W/cm^2^, cisplatin administered and 1.5 W/cm^2^ US treated group; CP + MEL, cisplatin administered and melatonin treated group. Data were represented as mean ± SEM. The degree of significance was symbolized by an asterisks (*) for the comparison of all groups with respect to the control group; by a double daggers (‡) for the comparison of the treatment groups with respect to the cisplatin administered group; by a daggers (†) for the comparison of ultrasound treated groups with respect to the melatonin administered group and by a section (§) for the comparison of 1 MHz 1.5 W/cm^2^ US treated group with respect to the 1 MHz 0.5 W/cm^2^ US treated group. (*p < 0.05, **p < 0.01, ***p < 0.001).

Figure 3a demonstrates the malondialdehyde (MDA) levels and superoxide dismutase (SOD) activity in sciatic nerves. The amount of MDA, a product of lipid peroxidation, was observed to be increased in nerve homogenates of cisplatin-induced neuropathy group (*p* < 0.01) in comparison to that of control, which indicates the increase in oxidative stress^20^. Upon treatments, MDA levels were observed to be significantly decreased in comparison to that of cisplatin-induced neuropathy group (*p* < 0.05). In addition, the activity of SOD was observed to be significantly decreased in cisplatin administered group (*p* < 0.001), which was noted to be significantly increased in treatment groups in comparison to that of cisplatin administered group (*p* < 0.05). Increase in SOD activity in treatment groups indicated a decreased oxidative stress^20^, which is in accordance with the finding of decreased MDA levels in treatment groups.

In order to comment on the effect of treatments on inflammation, the levels of pro-inflammatory cytokines interleukin-1 beta (IL-1β) and interleukin-6 (IL-6) in serum samples of the experimental groups were assessed. As seen in Fig. 3b, there was a slightly higher content of IL-1β and IL-6 in the cisplatin administered group compared to the control group, although the increase was statistically insignificant. In the US treatment groups, the levels of these cytokines were observed to be decreased compared to the cisplatin administered group, where the decrease was more prominent in the US treatment groups (*p* < 0.05). IL-1β and IL-6, which are pro-inflammatory cytokines, are involved in the formation of inflammatory changes and the emergence of a rapid immune response. The results revealed that an immune response emerged in the cisplatin induced neuropathy group, which was ameliorated upon US treatments.

### Histological analysis

**Figure 4:**
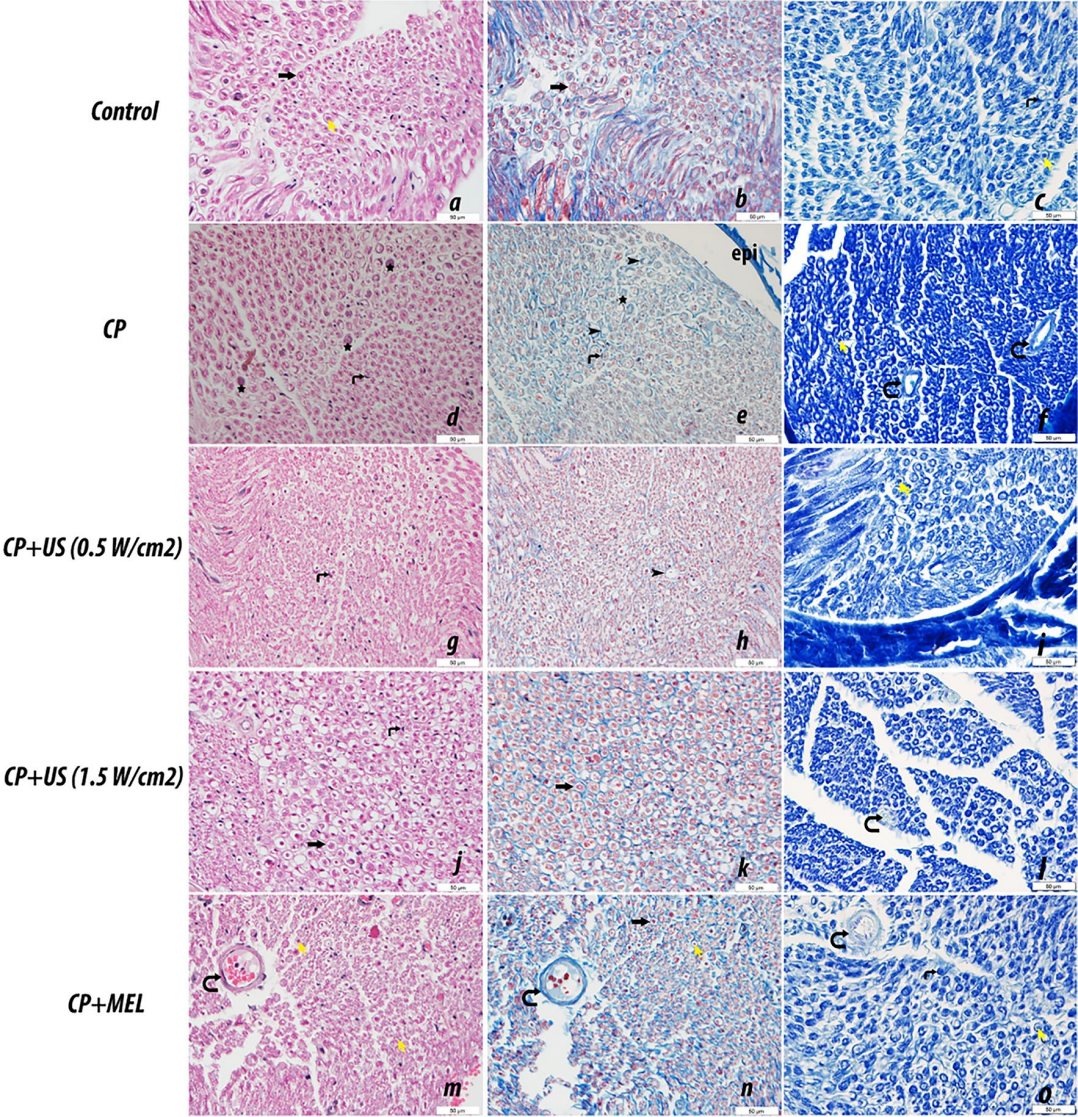
Hematoxylin–eosin staining (**a**, **d**, **g**, **j**, **m**), Masson’s trichrome staining (**b**, **e**, **h**, **k**, **n**) and luxol fast blue (LFB) staining (**c**, **f**, **i**, **l**, **o**) were performed on sciatic nerve tissue sections and representative images of the results from each group are presented. Notable features are indicated on the figure as follows: myelinated nerve fiber (  ), epineurium (epi), axon degeneration ( ), axon ( ), Schwann cell nucleus ( ), nerve fiber degeneration ( ), blood vessel ( ). C, control group; CP, cisplatin administered group; CP + US 0.5 W/cm^2^, cisplatin administered and 0.5 W/cm^2^ US treated group; CP + US 1.5 W/cm^2^, cisplatin administered and 1.5 W/cm^2^ US treated group; CP + MEL, cisplatin administered and melatonin treated group. Magnification × 400, magnification bar = 50 μm.

Figure 4 demonstrates the representative images of hematoxylin–eosin, Masson’s trichrome and luxol fast blue (LFB) stained sciatic nerve sections belonging to the study groups. LFB staining was used to visualize myelin/myelinated axons in nerve sections^21^. Masson’s trichrome stain was used for the visualization of the connective tissue component of the nerve and general histological changes, and to comment on the degree of degeneration in nerve sections^22^. In the sciatic nerve sections of the control group, the nerve fibers had a normal appearance and the nerve fiber myelination was also at normal levels. However, focal areas of demyelination and degenerative changes in nerve fibers were detected in cisplatin administered group. Upon treatments, the areas of demyelination and degeneration were observed to be decreased. The LFB stained images were further analyzed using ImageJ program to calculate the axon diameter, myelin sheath thickness and myelinated fiber diameter in the experimental groups (Table 4). There was a significant decrease in myelin sheath thickness (*p* < 0.01) together with a significant decrease in axon diameter (*p* < 0.01) and myelinated fiber diameter (*p* < 0.001) in cisplatin administered group in comparison to that of control. Treatments, more prominently the US treatments, led to an increase in these calculated parameters (Table 4). Especially in the 1.5 W/cm^2^ US treatment group, the results were observed to be similar to that of control group (Table 4).

**Table 4:** The results of the alterations in axon diameter, myelin sheath thickness and myelin fiber diameter in the experimental study groups.

Measurements (μm)	Groups				
	C	CP	CP + MEL	CP + US (0.5 W/cm^2^)	CP + US (1.5 W/cm^2^)
Myelinated fiber diameter	2.64 ± 0.01	1.76 ± 0.01***	1.87 ± 0.02**^,‡^	2.29 ± 0.01**^,‡^	2.35 ± 0.01**^,‡^
Myelin sheet thickness	0.60 ± 0.00	0.37 ± 0.00**	0.42 ± 0.00*	0.46 ± 0.00^‡^	0.53 ± 0.00^‡^
Axon diameter	2.14 ± 0.01	1.60 ± 0.01**	1.78 ± 0.02	1.96 ± 0.01*^,‡^	2.03 ± 0.01*^,‡^

The data were represented as mean ± standard error of mean. Differences in between variances were compared by One-way ANOVA test with Tukey’s test applied as a post-hoc test. *P*-values equal or less than 0.05 were considered as statistically significant (*p < 0.05, **p < 0.01, ***p < 0.001). The degree of significance was symbolized by an asterisks (*) for the comparison of all groups with respect to the control group; by a double daggers (‡) for the comparison of the treatment groups with respect to the cisplatin administered group; by a daggers (†) for the comparison of ultrasound treated groups with respect to the melatonin administered group and by a section (§) for the comparison of 1 MHz 1.5 W/cm^2^ US treated group with respect to the 1 MHz 0.5 W/cm^2^ US treated group.

## Discussion

The present study was conducted to assess the therapeutic effects of low-intensity pulsed low-frequency US applied at two different power intensities on a rat model of cisplatin-induced neuropathy. Peripheral neuropathy induced by the administration of chemotherapeutic agents is a complication that frequently occurs in chemotherapeutic intervention. Platinum compounds (cisplatin, carboplatin, oxaliplatin) belonging to the class of neurotoxic chemotherapeutic agents are associated with neuropathy. The symptoms progress to more proximal regions as the taken dose amount increases cumulatively^23^ or the duration of neuropathy can be affected. Therefore, animal models of agent-induced neuropathy have been used to advance treatment alternatives that can be applied to prevent or minimize nerve damage^24,25^. Cisplatin is one of the most widely used drugs to treat various cancers and is highly effective^26,27^. Due to its cytotoxic nature, cisplatin binds to nuclear DNA and activates some signaling pathways by disrupting DNA transcription and replication^27^. The rats in the current study received a weekly dose of 3 mg/kg i.p. injection of cisplatin for five weeks (a cumulative dose of 15 mg/kg cisplatin). This regime was chosen as it was previously reported to yield a cisplatin-induced neuropathy^24,28,29^ with a better clinical status of rats, and in addition, in order to create a similar course of administration as it is in human subjects.

Regardless of the advanced diagnosis and surgical treatments, patients receiving chemotherapeutic intervention may encounter permanent or sustained nerve damage. Although therapeutic US has a wide range of application areas in medicine and numerous studies reported the effects of US in nerve regeneration and growth in nerve injuries; only a few studies have been performed to examine the role of US in treatment of peripheral neuropathy. Previously, Daeschler et al*.* have reported that US therapy between 200 and 500 mW/cm^2^ applied after nerve injury significantly accelerated axonal regeneration and re-innervation of the muscle in nerve injury model, increased the number and myelination of distant axons to the lesion site, and improved nerve conduction velocity in the lowest intensity range of 200–300 mW/cm^2^^30^. Two studies in literature have compared the effects of low-level laser therapy and low-frequency (1 MHz) low-intensity US application in a pulsed mode in type-2 diabetic patients with carpal tunnel syndrome^31^ and in a continuous mode in patients with ulnar neuropathy at the elbow^32^, at 1 W/cm^2^ and 1.5 W/cm^2^ power intensities, respectively. Both of these studies have reported the beneficial effects of US application on clinical and electrophysiological parameters; however, no data to elucidate the mechanism of action of low-intensity US treatment in neuropathy were reported. In this current study, the results revealed the effectiveness of 1 MHz 0.5 and 1.5 W/cm^2^ pulsed US treatment on the repair of the nerve damage caused by cisplatin-induced neuropathy, and our findings are important for the further clinical application of US therapy and clarification of its mechanisms. Peripheral neuropathy was reported to result in pathological changes in the nerve, loss of deep tendon reflex, motor weakness, loss of myelinated fibers and decreased myelinated axon diameter^2^. Indeed, the results of this current study revealed a decrease in motor nerve conduction velocity together with histological changes in sciatic nerves, such as a decrease in myelin sheet thickness, myelinated fiber and axon diameters in cisplatin-induced neuropathy. Moreover, the decrease in CMAP amplitudes together with the slightly prolonged distal latency and CMAP duration may further indicate the axonal loss, damage and demyelination^17,18,33^ in sciatic nerves upon cisplatin administration. There was also a decrease in nociceptive pain perception in cisplatin administered group; therefore, the results of this study revealed a decrease in nerve function upon cisplatin administration. Low-intensity pulsed low-frequency US treatment applied at 0.5 and 1.5 W/cm^2^ power intensities significantly improved the nerve conduction velocities, CMAP amplitudes and decreased CMAP duration values together with nociceptive latencies. These findings are in accordance with the literature, as there are studies reporting the beneficial effects of US stimulation to increase nerve conduction velocities^34^, CMAP amplitude and area^35^ in nerve injury models. Savernini et al*.* have stated that the application of 0.3 and 0.4 W/cm^2^ therapeutic US led to the decrease in hyperalgesia induced by the chronic constriction injury on the infraorbital nerve of rats^36^. However, they have reported that therapeutic ultrasound did not alter the thermonociceptive threshold of sham and naive rats; therefore, they have suggested that the anti-nociceptive effects of therapeutic US could be triggered through the stimulation of macrophages and mast cells to secrete mediators in nerve injury^36^. Similarly, Chen et al*.* have observed that low-intensity pulsed US treatment attenuated lipopolysaccharide-induced neuroinflammation in the cortex and hippocampus of a mice model of neuroinflammation and memory impairment^37^. However, their results revealed that low-intensity US treatment resulted in dissimilar alterations when applied on sham or lipopolysaccharide-treated mice^37^; suggesting that the action of ultrasound may differ when there is an inflammatory stimulus present in the system. Supporting this notion, 0.5 W/cm^2^ US was reported to be sufficient to accelerate peripheral nerve regeneration in crush injury and the damaged nerve was reported to be more sensitive to US than the intact nerve^38^. In addition, in the current study, histological examinations revealed that myelin sheath thickness together with axon and myelinated fiber diameter was increased upon the US treatments in both power intensities. Therefore, low-intensity pulsed low-frequency US treatment ameliorated the nerve dysfunction observed in cisplatin administration, and the increase in nerve conduction velocity may be associated with apparent axonal healing^39^.

Nerve dysfunction in peripheral neuropathy is known to be associated with oxidative stress and neuro-inflammation. Peripheral nerves are susceptible to oxidative stress, which results in lipid peroxidation, neuroinflammation and damage to the myelin sheath due to weak cellular antioxidant defenses^40^. The results of the current study revealed the increase in pro-inflammatory cytokines and oxidative stress as a result of cisplatin administration, which were observed to be decreased upon US treatments. Nerve injuries result in the rapid production and secretion of pro-inflammatory cytokines, but their levels start to attenuate approximately after 7 days, and then anti-inflammatory cytokines are predominantly released^41^. However, Ruohonen et al*.* have demonstrated the prolonged inflammatory mediator changes taking place in a chronic denervation injury model of the sciatic nerve and have observed a marked increase in the expression of IL-1β levels in the epi-/perineurium and endoneurium 14 days after the injury together with the increase in number of macrophages in these regions^42^. Their findings support the observed increase in IL-1β levels observed in this current study upon cisplatin administration. Low-intensity pulsed US has been suggested to play a protective role in neuroinflammation by decreasing the expression levels of pro-inflammatory cytokines in the cortex of lipopolysaccharide (LPS)-treated mice model of neuroinflammation and memory impairment^37^ and in LPS-treated microglial cells^43^ through the modulation of toll-like receptor 4 (TLR4)/ nuclear factor kappa B (NF-κB) signaling. In addition, low-intensity focused US was reported to improve cerebral blood flow and to reduce neuroinflammation and pro-inflammatory cytokine levels by the inhibition of TLR4/NF-κB pathway in the medial prefrontal cortex of a rat model of vascular dementia^44^. Furthermore, Ito et al*.* have demonstrated that 1 MHz 140 mW/cm^2^ US stimulation led to a decrease in the gene expression levels of pro-inflammatory cytokines in sciatic nerves of a rat crush injury model^45^. They have suggested that US stimulation facilitated Wallerian degeneration, which in turn promoted early nerve regeneration, through the modulation of the levels of pro-inflammatory cytokines^45^. Therefore, the observed beneficial effects of low-intensity pulsed low-frequency US treatment on the improvement of sciatic nerve conduction might be related to the decrease in neuroinflammation, which was previously reported to increase the survivability of the neurons and synaptic functions^44^. However, the effects of US treatment on the levels of inflammatory cytokines together with the related signaling pathways needs further validation in future studies.

Oxidative stress and inflammation have important effects on organelle function. Nowadays, especially inflammation or an ongoing inflammation is thought to be the main cause of many diseases^46^. Increased oxidative stress causes damage to proteins and organelles, especially to the mitochondria. In addition, oxidative stress and increased inflammation may cause the activation of apoptotic or mitophagic pathways^47^. Members of the Bcl-2 family, consisting of pro-apoptotic and anti-apoptotic proteins, contribute to the survival or death of cells^48^. Disruption of the balance created by the members of Bcl-2 family facilitates the release of cytochrome c by altering the integrity of mitochondria and leading to the activation of apoptosis. Likewise, the impairment of calcium release or improper processing of proteins within the endoplasmic reticulum can lead to induction of apoptotic cell death^49^. Caspases, which are cysteine proteases, play a role in the course of apoptosis. In order for apoptosis to start in the cell, caspase-3 protein must be activated by the cleavage of procaspase-3, which in turn triggers apoptosis. Stimulation of apoptosis causes fewer side effects and is a preferred immune system cell death method^50^. In the current study, the expression level of uncleaved caspase-3 was observed to be increased in the cisplatin administered group and was decreased upon treatments, especially in the US treatment groups. In addition, the amount of anti-apoptotic Bcl-2 protein expression was significantly reduced upon cisplatin administration, which was increased to that of control levels by the treatments. Cisplatin was previously reported to induce apoptosis in neuronal cells^51^ and overexpression of Bcl-2 was shown to block cisplatin-induced apoptosis in a rat neuroblastoma cell line^48^. Therefore, the observed increase in pro-caspase-3 together with the decrease in anti-apoptotic Bcl-2 protein levels may indicate the apoptotic activation upon cisplatin administration. The low-intensity pulsed low-frequency US treatments in both power intensities restored the levels of these proteins, revealing that US treatment played a role in the regulation of apoptosis in sciatic nerves and suppressed apoptosis.

Pathological diseases such as inflammation, cancer, neuropathic and neurodegenerative diseases have been associated with mitophagy disorders. Parkin, the signal protein specific to mitophagy, can initiate the commissioning of the autophagy mechanism and facilitate the ingestion of damaged mitochondria into autophagosomes^52^. Therefore, the increase in the Parkin level is an indication for the activation of mitophagy pathway, which can be an effective mechanism to eliminate the damaged mitochondria. However, incomplete mitophagy is also harmful, as the neurons rely on mitochondrial respiration and the effects of deprived mitochondria cannot be diluted by cell division. As a result, damage to the mitochondria and other cellular components leads to constant disruption in neuronal functions and peripheral neuropathy^53^. For this reason, the decrease in Parkin levels observed in the cisplatin administered group in our study implied that the mitochondria damaged by oxidative stress may not be cleared sufficiently with mitophagy in cisplatin administration. However, the observed increase in Parkin levels in the 1.5 W/cm^2^ US group indicated that US treatment can regulate mitophagy and therefore it can be an important alternative in the elimination of nerve damage by the regulation of intracellular apoptotic and mitophagic pathways.

Melatonin was previously demonstrated to decrease lipid peroxidation, axonal injury and myelin breakdown in crush injuries^54^, prevent mitochondrial dysfunction and promote neuroprotection in oxaliplatin-induced neuropathy^40^ and in paclitaxel-induced neuropathic pain models^55^. Therefore, in this current study, melatonin administration was chosen as a positive control in order to compare the results of US treatments. In all of the parameters investigated, melatonin and US treatments yielded similar results. However, low-intensity pulsed low-frequency US treatment was observed to be more effective especially in activating mitophagy, suppressing apoptosis, decreasing inflammation and reducing demyelination and nerve degeneration in sciatic nerves after cisplatin administration.

Taken together, low-intensity pulsed low-frequency US treatment ameliorated the nerve dysfunction in cisplatin-induced peripheral neuropathy by decreasing oxidative stress and inflammation, regulating apoptosis and mitophagy, reducing demyelination and nerve degeneration, thereby increasing motor nerve conduction and nociceptive pain perception. However, there are some limitations of this study. The major limitation is that the levels of only two pro-inflammatory markers and the expression levels of only one protein in the apoptosis and mitophagy pathways were considered. Therefore, the role of US treatment particularly in modulating apoptosis, mitophagy and mitochondrial dysfunction as well as inflammatory pathways, as implied by the results of the current study, should be more clearly addressed in future studies. In this study, the alterations in the expression level of uncleaved caspase 3 were evaluated, however, in order to better comment on the modulation of apoptosis upon US stimulation, the expression levels of cleaved caspase 3 together with other caspases in the cascade should be assessed in future studies. The levels of pro-inflammatory cytokines were measured using ELISA tests, which might have given less sensitive results; therefore, the alterations in inflammatory markers should be determined in future studies using more sensitive techniques. In addition, US applied control groups were not included in the study, which prevented the consistent comparison of the effects of US treatments, especially in the interpretation of the results of nociceptive latencies and levels of pro-inflammatory cytokines. Consequently, low-intensity pulsed low-frequency US therapy can be considered as an alternative strategy for the treatment of chemotherapy-induced neuropathies and warrants further investigations.

## Materials and methods

### Animals

All experimental procedures used in this study were approved by the Ethics Committee of Aydın Adnan Menderes University with an approval number 64583101/2019/012 and were performed in accordance with the ARRIVE guidelines. All experimental methods were performed in accordance with relevant guidelines and regulations. Adult male Wistar-albino rats (*n* = 50) weighing 300–350 g were kept in an environment temperature of 22 ± 1˚C, 12/12 h of light/dark cycle, relative humidity (40–50%) and air conditioning controlled semi-climatized laboratory conditions. The rats were randomly divided into 5 experimental groups (10 animals in each group) as control (C), cisplatin administration (CP), 10 mg/kg melatonin treatment (CP + MEL), 1 MHz frequency 0.5 W/cm^2^ pulsed US treatment (CP + US 0.5 W/cm^2^) and 1 MHz frequency 1.5 W/cm^2^ pulsed US treatment (CP + US 1.5 W/cm^2^) groups. The rats in the control group only received vehicle and the rats in the other groups were administered with 3 mg/kg cisplatin injection once a week for a 5-week period for the induction of chemical neuropathy. Afterwards, melatonin and low-intensity pulsed low-frequency US treatments were performed for 15 consecutive days (Fig. 1).

### Drugs and their administration

In order to achieve a rat model of cisplatin-induced neuropathy, cisplatin (Item no: 13119, Cayman Chemical, Michigan, USA) was dissolved in saline and was intraperitoneally (i.p.) administrated at the dose of 3 mg/kg once a week for five weeks (total cumulative dose is 15 mg/kg), as previously reported in the literature^24,28,29^. In order to prevent kidney injury due to cisplatin injection, the calculated dose was mixed with 2 ml of physiological saline prior to injection. Melatonin (Item no: 14427, Cayman Chemical, Michigan, USA) was dissolved in 5% ethanol and was administered to the rats in CP + MEL group intraperitoneally at a dose of 10 mg/kg/day for 15 consecutive days.

### Low-intensity pulsed low-frequency US treatment

Low-intensity pulsed low-frequency US was applied to the rats with cisplatin-induced chemical neuropathy by BTL5710 SONO US device (BTL Medical Technologies, Czechia). The rats were restrained in prone position and their head was covered with a cloth, enabling them to keep the same position for 3 min. The US was applied onto the dorsolateral region of the hind-limb for 3 min onto an approximately 5 cm^2^ skin area above the sciatic nerve on the lateral thigh of each leg. The US treatment was applied to both legs (right and left leg) at 1 MHz frequency and a power density of 0.5 W/cm^2^ and 1.5 W/cm^2^ in a pulsed mode at 20% duty cycle for 15 days^32^*.*

### Nociceptive tests

Rats in the study groups were subjected to nociceptive tests, hot plate and tail flick tests, on 5th, 19th and 33th days of cisplatin administration period and once a week during the treatment period. For the tail-flick test, a semi-automatic device with 8 V / 50 W radiant heat source (tail-flick device, May Tic., Ankara, Turkey) was used. The heater source was applied to the lower 1/3 of the tail of all rats. The movement of tails were automatically detected by the device and the heat stimulus was interrupted, the latency for flicking the tails was recorded. In order to prevent permanent damage to the tail, the cut-off time was determined as 10 s. For the hot-plate test, a preheated (55 ± 0.3°C) (circular metal surface (Hot plate device, May Tic., Ankara, Turkey) was used. Response times to the thermal stimulation was recorded, such as the time for licking the hind legs or jumping out of the device. The cut-off time was considered as 15 s to prevent any damage. The test was repeated 3 times with an interval of 10 min and the average time was used in data analysis.

### Electrophysiological measurements

The electrophysiological measurements were performed after 15 days of treatment. The rats were anesthetized using an intraperitoneal injection of ketamine and Xylazine (70:10 mg of ketamine: Xylazine per kg). Afterwards, the left lateral femoral region was shaved and sciatic nerves were exposed through a curvilinear surgical incision. The sciatic nerve was placed onto the in vivo electrodes (Biopac Systems Inc., USA) and a supramaximal electrical stimulation was applied on the sciatic nerve via in vivo distal and proximal electrodes fixed at a distance of 1.1 cm connected to the Biopac MP100 system (Biopac Systems Inc., USA). The recording needle electrodes were placed in the second interosseous cavity of the hind foot and were used to record the CMAP. The ground electrode was placed at the lateral side of the hind foot. The electrical stimulation (0.1 ms, 1 Hz, 7 mV) was applied through the distal and proximal electrodes, respectively, and the resulting CMAPs were recorded from the recording needle electrode and transferred to the computer with the aid of an amplifier. The obtained potentials were analyzed with AcqKnowledge data acquisition and analysis software (Biopac Systems Inc., USA) to record distal and proximal latencies, the duration of CMAP, CMAP peak-to-peak amplitudes and the area under this curve. In addition, MNCV was calculated via the following formula:

MNCV = Distance between distal and proximal electrodes / (Proximal latency—distal latency).

After the collection of electrophysiological recordings, the rats were sacrificed and blood samples were collected. The sciatic nerves dissected from the left leg were stored at − 80℃ and were used for biochemical measurements and Western blot analysis. The sciatic nerves dissected from the right leg were placed into 10% formalin and were used in the histological analysis.

### Western blot analysis

For western blot assay, sciatic nerves were homogenized in a RIPA lysis buffer (Catalog # 632424, Merck Millipore, Darmstadt, Germany) including 0.5 M Tris–HCl, pH 7.4, 1.5 M NaCl, 10 µg/ml aprotinin, 1 mM PMSF, 2.5% deoxycholic acid, 10% NP-40, 10 mM EDTA and 1:10 protease inhibitor cocktail (Catalog # P8340, Sigma-Aldrich, USA) was added into the buffer. Then lysates were centrifuged at 15.000 g for 10 min at 4 ℃, and the supernatants were collected. Tissue lysates were mixed with sample buffer including 0.5 M Tris–HCl (pH 6.8), glycerol, %10 SDS, bromophenol blue and β-mercaptoethanol, and boiled at 95 ^0^C for 5 min. The protein concentration in lysates was determined by using bicinchoninic acid protein assay kit (Catalog # 23227, ThermoFischer Scientific, USA). An equal amount of protein (20 μg) was taken from each sample and loaded onto polyacrylamide gel (containing 4 and 12% stacking and resolving gels, respectively), subjected to gel electrophoresis and then transferred onto polyvinylidene fluoride membranes (Catalog # IPVH00010, Millipore, Bedford, MA, USA). After blocking, the membranes were incubated with primary antibodies against Parkin (1:1000, Catalog # sc-32282, Santacruz, USA), caspase-3 (1:1000, Catalog # sc-56052, Santacruz, USA), Bcl-2 (1:1000, Catalog # sc-7382, Santacruz, USA) and β-actin (1:1000, Catalog # sc-47778, Santacruz, USA) at 4℃ overnight. After washing, the secondary antibody (1:2000, anti-mouse IgG HRP, Catalog # 7076S, Cell Signaling, USA) was applied at room temperature for 2 h, and then the membrane was washed, ECL substrate (Catalog #1705060, BIO-RAD, California, USA) was added and finally membranes were visualized using UVP system (LAB Denmark APS, Denmark) with the same exposure time of 5 min. Measurement of the protein band intensities was performed using ImageJ (MD, USA, 64 bit version) software, as described in the ImageJ user guide. For the quantification of the band intensities, no image processing was performed. The bands corresponding to a specific protein of each experimental group were selected as region of interest and analyzed separately.

### Biochemical analysis

The malondialdehyde (MDA) levels and superoxide dismutase (SOD) activity in sciatic nerve tissues were measured by the use of appropriate measurement kits (Catalog # 10009055 for MDA and Catalog # 706002 for SOD assay kit, Cayman Chemical, Michigan, USA) according to the manufacturer's instructions. Likewise, the levels of serum interleukin 1 β (IL-1β) and interleukin 6 (IL-6) levels were determined using appropriate Elisa kits (Elabscience, USA) according to the instructions of the manufacturer (Catalog # E-EL-R0012 for IL-1β and Catalog # E-EL-R0015 for IL-6 Elisa kits).

### Histological analysis

The dissected right sciatic nerves were fixed in 10% formaldehyde. After the removal of formaldehyde with running water, the tissues were dehydrated through passages from a series of ethanol, infiltrated with paraffin and were embedded in paraffin blocks (Catalog # TK2050066, Tekkim, Turkey) as described previously^22,24^. 5 μm thick sections were obtained with a rotary microtome (Leica RM 2265, Germany) using a Plasma LS35 Microtome blade. The sections were then stained using Hematoxylin–Eosin, Luxol fast blue (LFB) and Masson’s trichrome staining kits (Atom Scientific, UK) according to the instructions of the manufacturer (Catalog # RRSK26-100 for Hematoxylin-Eosin stain kit, Catalog # RRSK20-100 for Masson’s trichrome stain kit and Catalog # RRSK345-100 for LFB stain kit). The stained sections were photographed using an Olympus DP73 microscope (Olympus, Japan). The LFB stained images were further analyzed for the measurements of myelin sheet thickness, axon and myelinated fiber diameter by the use of ImageJ (National Institute of Health, USA) program.

### Statistical analysis

The data were analyzed with SPSS version 22 for Windows (IBM, USA), and were represented as mean ± standard error of mean (SEM). Statistical analysis of the differences in variance was analyzed using one-way analysis of variance (one-way ANOVA) followed by Tukey’s test as a post-hoc test. A p value equal to or less than 0.05 was accepted as statistically significant (*p < 0.05, **p < 0.01, ***p < 0.001). The degree of significance was symbolized by an asterisks (*) for the comparison of all groups with respect to the control group; by a double daggers (‡) for the comparison of the treatment groups with respect to the cisplatin administered group; by a daggers (†) for the comparison of ultrasound treated groups with respect to the melatonin administered group and by a section (§) for the comparison of 1 MHz 1.5 W/cm^2^ US treated group with respect to the 1 MHz 0.5 W/cm^2^ US treated group.

## Supplementary Information

Supplementary Information.

## Data Availability

The datasets generated in the current study are available from the corresponding author on request.
