# Supplementary material for: Low-intensity low-frequency pulsed ultrasound ameliorates sciatic nerve dysfunction in a rat model of cisplatin-induced peripheral neuropathy
Source: Sci Rep. 2022 May 17;12:8125. doi: 10.1038/s41598-022-11978-z (PMC9114430; doi:10.1038/s41598-022-11978-z)
Supplement: Supplementary file 1 — Supplementary Information. [file 41598_2022_11978_MOESM1_ESM.pdf]

Supplementary Information for Bilir-Yildiz et al. “Low-intensity low-frequency pulsed ultrasound ameliorates sciatic nerve dysfunction in a rat model of cisplatin-induced peripheral neuropathy”

Busra Bilir-Yildiz, Fatma Bahar Sunay, Hatice Fulya Yilmaz, Ozlem Bozkurt-Girit

The Western blot images represented in Figure 2 in the manuscript were cropped from different blots for the visualization of different protein expression levels, namely β-actin, Parkin, bcl-2 and caspase-3. Each blot used for the determination of protein expression analysis, was visualized under same exposure time of 5 min. The full-length blots can be seen below in the Supplementary Figure S1.

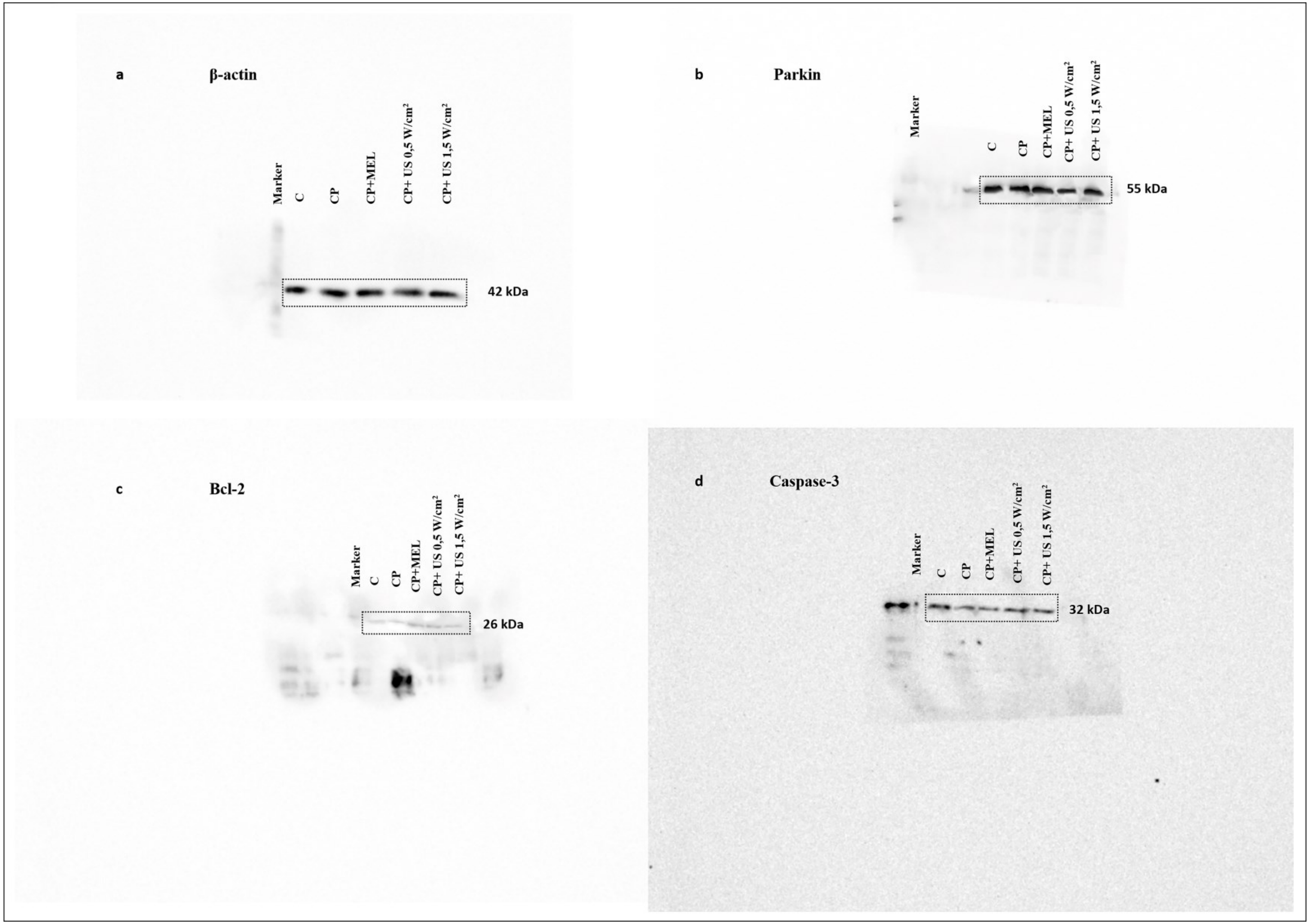
a
β-actin
Marker
C
CP
CP+MEL
CP+ US 0,5 W/cm²
CP+ US 1,5 W/cm²
42 kDa
b
Parkin
Marker
C
CP
CP+MEL
CP+ US 0,5 W/cm²
CP+ US 1,5 W/cm²
55 kDa
c
Bcl-2
Marker
C
CP
CP+MEL
CP+ US 0,5 W/cm²
CP+ US 1,5 W/cm²
26 kDa
d
Caspase-3
Marker
C
CP
CP+MEL
CP+ US 0,5 W/cm²
CP+ US 1,5 W/cm²
32 kDa

Supplementary Figure S1. **The full-lenght Western blot images of the results of Wes[illegible]n blot images demontrated in Figure 2 in the manuscirpt**. Western blots of a) β-actin, b) Parkin, c) bcl-2, d) caspase-3. The same exposure was used to visualize the blots. The cropped parts for the representation in Figure 2 were marked on the images. The samples were derived from the same experiment and the blots were processed in parallel. C, control group; CP, cisplatin administered group; CP+US 0.5 W/cm^2, cisplatin administered and 0.5 W/cm^2 US treated group; CP+US 1.5 W/cm^2, cisplatin administered and 1.5 W/cm^2 US treated group; CP+MEL, cisplatin administered and melatonin treated group.
